# Hand Grip Strength Index, a Novel Tool in Risk‐Assessing Multi‐Ethnic End‐Stage Kidney Disease Patients Treated by Haemodialysis

**DOI:** 10.1111/jhn.13414

**Published:** 2024-12-17

**Authors:** Mai Nguyen, Andrew Davenport

**Affiliations:** ^1^ UCL Centre for Kidney & Bladder Health, Royal Free Hospital, University College London London UK

**Keywords:** bioimpedence, frailty, haemodialysis, hand grip strength, mortality, sarcopenia

## Abstract

**Introduction:**

Increasing numbers of elderly co‐morbid patients with end‐stage kidney disease (ESKD) are now offered haemodialysis. Simple, rapid screening tools are required to risk‐assess patients, highlighting those requiring nutritional or other support and advising on prognosis. As such, we assessed a newly introduced tool, the hand grip strength index (HGS index), a comparison of measured to predicted HGS.

**Methods:**

We reviewed ESKD dialysis patients dialysing under the care of an inner‐city tertiary dialysis centre who had contemporaneous HGS measurements, and body composition measured by multifrequency bioelectrical impedance analysis, followed for ≤ 9 years, censoring for transplantation.

**Results:**

Results from 1023 patients, 63.2% male, 48.2% White and 46.5% diabetic, with a dialysis vintage of 21.2 (7.2–61.0) months, were analysed. Mortality was significantly greater for those in the lowest HGS index quartile using Kaplan–Meier analysis (*p* < 0.001). On multivariable step‐backward Cox regression analysis, mortality was independently significantly associated (*p* < 0.001) with increasing age (hazard ratio [HR] 1.04 95% confidence interval (CI) [1.029–1.045]), higher co‐morbidity score (HR 1.24 [1.142–1.347]) and post‐dialysis extracellular water/total body water ratio (HR 1.15 [1.089–1.219]) and lower HGS index (HR −0.76 [0.991–0.998]), whereas sarcopenia and frailty were not retained in the model.

**Conclusion:**

Increasing numbers of elderly co‐morbid patients are being treated with dialysis, so simple screening tools are required to advise on prognosis and highlight patients who may need additional support, including nutrition. We found the HGS index to have prognostic value, along with the traditional risk factors of patient age and co‐morbidity.

## Summary

- Increasing numbers of elderly frail patients with progressive chronic kidney disease are now being offered kidney dialysis treatment.
- Simple, rapid screening tools are required to risk‐assess patients, highlighting those requiring nutritional or other support and advising on prognosis.
- Compared to standard assessments of frailty, co‐morbidity and estimates of dietary protein, the hand grip strength index, compared to that predicted for age, height and gender, was associated with patient survival.

## 1 Introduction

The demographics of the haemodialysis population in economically developed countries have changed over time, with increasing numbers of older patients and increasingly co‐morbid patients with end‐stage kidney disease (ESKD) now being offered dialysis [1]. Dialysis patients are instructed to restrict their dietary intake of certain foodstuffs [2], which has led to concerns about nutritional intake, particularly for the older patient [3]. Although there is a physiological loss of muscle mass with ageing, patients with ESKD are recognised to be at greater risk of developing sarcopenia, a greater loss of muscle mass than that expected for age [4]. Current guidelines recommend cut‐off values for muscle mass, typically measured by dual‐energy x‐ray absorptiometry or bioelectrical impedance [5] and muscle strength using hand grip strength (HGS), or anthropometric methods [6] or assessments of physical activity [7, 8].

Loss of muscle mass and strength increases the risk of frailty, a multi‐dimensional, ageing‐related syndrome of physiological deterioration, associated with an inadequate response to health stressors and coupled with an increased susceptibility to illness and mortality due to weakness and vulnerability [9, 10]. Although dietary diaries, histories and food frequency questionnaires can be used to assess protein and other nutrient intake [11, 12], these rely on patient memory and compliance, so are often of limited value in an older co‐morbid patient population. More recently, the simplified creatinine index (SCI) has been introduced as an assessment of dietary protein intake and demonstrated to be a significant risk factor for mortality of haemodialysis patients [13].

More recent studies have reported that measurements of muscle mass in dialysis patients can be confounded by increased muscle water content, fat and fibrous tissue [14, 15], and as such, there has been greater interest in muscle function, which has led to the development of the HGS index, which compares the maximal patient HGS with that predicted for age and gender, with lower values associated with increased risk of mortality [16].

As such, we wished to examine the performance of these newer tools as potential risk indicators for haemodialysis patient mortality compared to some of the traditional biomarkers.

## 2 Patients and Methods

We reviewed the records of haemodialysis outpatients dialysing under the care of a tertiary inner‐city university hospital who had HGS recorded on the same day as bioimpedance measurements and standard laboratory investigations, between July 2014 and April 2021, with patients followed up to May 2024. Body composition and extracellular water (ECW)/total body water (TBW) ratios were assessed using multifrequency bioimpedance assessments (MFBIA) (InBody 720, or S10, Seoul, South Korea) following a standardised protocol, with measurements made post‐completion of the mid‐week dialysis session, after allowing time for re‐equilibration [5, 17]. To compare measurements of body composition between patients, lean body mass and appendicular lean mass were indexed to height squared. Sarcopenia was defined using the respective appendicular lean body mass index (ALMI) and HGS cut‐offs for both non‐Asian patients (< 7.0 kg/m^2^ and women < 5.5 kg/m^2^, and HGS < 27 kg for men and < 16 kg for women [7]) and Asian patients (ALMI for men < 7.0 kg/m^2^ and women < 5.7 kg/m^2^, and HGS < 28 kg for men and < 18 kg for women [8]), respectively. HGS was measured using a calibrated grip‐D strength dynamometer (Takei Scientific Instruments Co., Nigata, Japan). Patients were instructed according to the manufacturer's instructions on how to perform their maximal voluntary effort, the maximum value of three measurements in the dominant arm was recorded [18] and the predicted HGS and HGS index were then calculated [16]. Frailty was recorded using the 9‐point Canadian geriatric clinical frailty scale (CFS) [10, 19] adopted by the United Kingdom National Institute for Health and Care Excellence (NICE) for National Health Service (NHS) patients, defining frailty as a score of > 4.

Patient demographics, routine laboratory investigations and relevant medical histories were obtained from computerised medical records, and co‐morbidity was assessed using the UK Stoke–Davies grading [20]. Nutrition was assessed using both the geriatric nutritional risk index (GNRI) [21] and the SCI [13]. Dialysis prescriptions and session details were obtained from hospital computerised records and patients dialysed with Fresenius 4008H and 5008H dialysis machines (Fresenius Medical Company, Bad Homburg, Germany) or B‐Braun Dialogue D+ (B Braun, Melsungen, Germany) using high‐flux polysulphone dialysers and ultrapure quality dialysis water, with dialysis sessional urea clearance (Kt/V) calculated using standard equations.

### 2.1 Statistical Methods

Categorical data are presented as numbers (percentage) and continuous data are presented as mean ± standard deviation for normally distributed data or median (interquartile range) for non‐parametric data. Standard statistical tests were used to analyse data, including Chi‐square (*χ* ^2^) analysis for categorical data, and ANOVA or the Kruskal–Wallis test was used to compare numerical data between groups, with appropriate corrections made for multiple testing (Tukey and Games Howell). Association with mortality was analysed by receiver operator curves (ROCs), followed by Kaplan–Meier, and step backward multiple variable Cox regression was used to analyse patient survival censored in May 2024, with patients who underwent transplantation censored at the time of transplantation. Non‐parametric variables were log‐transformed or entered as categorical data as necessary. Models were checked for both collinearity and collider bias. Statistical analysis was carried out using Prism 10.2 (Graph Pad, San Diego, USA) and SPSS29 (IBM, Armonk, New York, USA). Statistical significance was considered at *p* < 0.05.

### 2.2 Ethical Approval

This retrospective audit followed the UK NHS guidelines for clinical audit and service development, and registered with the Royal Free Hospital audit department. development. As the audit complied with NHS guidelines (UK NHS guidelines for clinical audit and service development, available at http://www.hra.nhs.uk/documents/2013/09/defining-research.pdf, and http://www.gov.uk/government/publications/health-research-ethics-committees governance arrangements).

## 3 Results

We studied 1023 patients, 63.2% male, 48.2% White, 27.2% African‐Afro‐Caribbean and 24.6% Asian, and 46.5% diabetic, who had been established on regular dialysis for 21.2 (7.2–61.0) months (Table 1). In terms of additional co‐morbidity, 17% had past medical histories of myocardial infarction, 10.9% had peripheral vascular disease, 11.4% had cerebrovascular stroke and 16.3% had cancer. Two hundred and twenty‐nine patients (22.4%) had been prescribed one or more doses of prednisolone at some time point prior to study, and there was no difference between groups (20.7 vs. 23.1 vs. 19.2 vs. 26.5%). No patient was in receipt of anabolic steroids, of recombinant growth hormone, although patients were prescribed erythropoietin‐stimulating agents to manage anaemia secondary to ESKD. At the time of measuring HGS, 70 (6.8%) patients were prescribed steroids, median daily dose 5 mg (range < 2–20 mg), but again, there was no difference between groups (Table 2). The majority of patients, 592 (57.9%), were prescribed hydroxymethylglutaryl‐CoA reductase inhibitors (statins), and there were no differences between groups. No patient was in receipt of anabolic steroids, recombinant human growth hormone or glucagon‐like peptide‐1 receptor agonists.

**Table 1:** Patient demographics and relevant clinical history and dialysis sessional adequacy.

Variable (s)	Group 1 (*n* = 256)	Group 2 (*n* = 255)	Group 3 (*n* = 255)	Group 4 (*n* = 257)	*p* value
Age (years)	64.9 ± 15.6	64.2 ± 16.1	65.6 ± 15.4	63.8 ± 14.6	> 0.05
Male (%)	143 (55.9)	163 (63.9)	169 (63.3)	172 (66.9)*	0.036
White/African/Asian (%)	45.1/33.9/30.9	51.3/28.9/29.8	54.4/26.7/28.9	61.2/30/17.1*	0.013
Dialysis vintage (months)	26.1 (9.4–75.5)	24.1 (7.3–61)	18.8 (5.8–56)	18.2 (5.3–47.3)	> 0.05
Weight (kg)	70.7 ± 18.5	70.4 ± 17.8	70.2 ± 17.9	74.6 ± 18.2*	0.047
Body mass index (kg/m^2^)	26.3 ± 5.8	25.3 ± 5.7	25.5 ± 5.7	27.4 ± 6.2	> 0.05
MAP (mmHg)	90.0 ± 17.3	90.1 ± 19.3	93.1 ± 17.7	93.4 ± 16.7	> 0.05
Diabetic (%)	139 (54.7)	127 (49.8)	115 (45.1)*	93 (36.5)***	< 0.001
History MI/PVD (%)	20.9/11.8	16.1/13.3	16.9/9.4	14.1/9.0	> 0.05
History CVA/cancer (%)	16.1/11.0	12.2/14.5	9.0/20.0	28.2*/16.3	0.021
Davies co‐morbidity	2 (1–3)	2 (1–2)	1 (1–2)**	1 (1–2)***	< 0.001
Clinical frailty score	5 (4–6)	4 (3–6)***	4 (3–5)***	4 (3–4)***	< 0.001
Frail (%)	155 (60.5)	123 (48.2)*	106 (41.6)*	63 (24.5)***	< 0.001
Sarcopenia HGS (%)	256 (100)	234 (91.8)***	159 (62.4)***	55 (21.4)***	< 0.001
Sarcopenia ALMI (%)	92 (36.5)	92 (37.1)	91 (36.3)	56 (25.5)***	< 0.001
Sarcopenia (%)	92 (36.5)	87 (35.2)	76 (30.3)	24 (9.4)***	< 0.001
Dialysis session (h)	3.6 ± 0.5	3.6 ± 0.6	3.6 ± 0.6	3.5 ± 0.6	> 0.05
Dialyser surface area (m^2^)	1.9 ± 0.3	1.9 ± 0.3	1.9 ± 0.3	1.9 ± 0.3	> 0.05
Urea reduction ratio (%)	72.7 ± 9.0	71.9 ± 9.2	71.4 ± 10.0	70.1 ± 9.9*	0.02
Dialyser (Kt/Vurea)	1.56 ± 0.42	1.51 ± 0.37	1.82 ± 0.48*	1.44 ± 0.4*	0.01
Transplanted (%)	35 (13.7)	54 (21.2)*	52 (20.4)*	84 (32.7)***	< 0.001
All deaths (%)	168 (65.6)	161 (63.1)	138 (54.1)**	129 (50.2)***	< 0.001
Censored deaths (%)	165 (64.5)	152 (60)	130 (51.0)*	116 (45.1)***	< 0.001

*Note:* Patients were divided into quartiles according to hand grip index from lowest (group 1) to highest (group 4). Statistical analysis *p < 0.05, **p < 0.01, ***p < 0.001 versus group 1, and *p* values group 1 versus group 4. Variables expressed as integer, percentage, mean ± SD and median (interquartile range), patients censored at the time of transplantation (censored deaths). Past medical history MI, PVD, CVA, patients meeting ALMI, HGS criteria for sarcopenia and meeting both criteria (sarcopenia).

Abbreviations: ALMI, appendicular lean mass index; Ethnicity African, Afro‐Caribbean; CVA, cerebrovascular accident; HGS, hand grip strength; MAP, mean arterial blood pressure; MI, myocardial infarction; PVD, peripheral vascular disease.

**Table 2:** Patient strength and body composition and relevant laboratory investigations.

Variable (s)	Group 1 (*n* = 256)	Group 2 (*n* = 255)	Group 3 (*n* = 255)	Group 4 (*n* = 257)	*p* value
Hand grip strength (kg)	9.6 (7.0–13.2)	16.3 (12.8–21)***	20.6 (17–26.1)***	27.8 (22.2–35.4)***	< 0.001
Hand grip Index	42.2 (33.4–50.1)	65.5 (59.7–70.8)***	82.2 (77.1–88.7)***	108.6 (99.2–121.7)***	< 0.001
FFMI (kg/m^2^)	17.2 ± 3.3	17.3 ± 2.8	17.5 ± 2.9	18.5 ± 2.8***	< 0.001
SLMI (kg/m^2^)	16.2 ± 3.3	16.3 ± 2.7	16.5 ± 2.8	17.4 ± 2.7***	< 0.001
SMMI (kg/m^2^)	9.2 ± 2.2	9.3 ± 1.6	9.4 ± 1.7	10.0 ± 1.7***	< 0.001
ALMI (kg/m^2^)	7.1 ± 2.3	7.2 ± 1.8	7.1 ± 1.5	7.6 ± 1.9**	0.002
Canaud LTMI (kg/m^2^)	18.7 (15.6–22.5)	18.9 (16.1–22.5)	19.1 (15.9–22.4)	22.1 (17.5–25)***	< 0.001
% Body fat	31.0 ± 12.2	29.7 ± 12.0	28.8 ± 12.0	31.7 ± 21.1	> 0.05
ECW/ht pre‐HD (L/m)	8.9 ± 2.1	9.0 ± 1.8	9.0 ± 1.9	9.5 ± 1.8*	0.014
% ECW/TBW pre‐HD	40.5 ± 4.0	40.5 ± 1.6	40.1 ± 2.9	40. ±1.5	> 0.05
ECW/ht post‐HD (L/m)	8.5 ± 2.1	8.5 ± 1.9	8.6 ± 1.7	8.9 ± 1.8*	0.034
%ECW/TBW post‐HD	39.8 ± 3.4	39.9 ± 1.8	39.6 ± 1.8	39.3 ± 1.7*	0.039
BMR (kcal/day)	1406 ± 269	1420 ± 258	1430 ± 252	1470 ± 276*	0.042
SCI (mg/kg/day)	17.7 (16.2–19.5)	17.9 (16.4–20.3)	18.1 (16.7–20.1)	18.5 (16.8–21.1)	> 0.05
GNRI	102 (94–113)	102 (94–111)	104 (96–117)	109 (102–127)	> 0.05
Major/moderate/low/no risk (%)	8.2/12.1/17.3/62.5	8.2/12.5/14.5/64.5	3.5/12.5/15.7/68	2.3/4.3/8.2/85.5***	< 0.001
Haematocrit (%)	34.3 ± 4.8	34.0 ± 4.5	34.0 ± 4.6	33.9 ± 4.4	> 0.05
Urea (mmol/L)	20.1 ± 10	19.7 ± 6.7	19.8 ± 6.7	20.5 ± 6.6	> 0.05
Creatinine (μmol/L)	626 (490–798)	687 (520–830)	682 (564–860)	710 (581–908)	> 0.05
Albumin (g/L)	38 (35–41)	38 (35–41)	39 (35–41)	40 (37–42)	> 0.05
C‐reactive protein (g/L)	7 (2–17)	7 (3–17)	5 (2–13)	6 (2–14)	> 0.05
Prescribed steroid (%)	15 (5.9)	24 (9.4)	15 (5.9)	16 (6.2)	> 0.05
Prescribed statin (%)	160 (63)	151 (59.2)	143 (56.1)	138 (58.1)	> 0.05

*Note:* Patients were divided into quartiles according to hand grip index from lowest (group 1) to highest (group 4). Statistical analysis *p < 0.05, **p < 0.01, ***p < 0.001 versus group 1, and *p* values group 1 versus group 4. Variables expressed as integer, percentage, mean ± SD or median (interquartile range).

Abbreviations: ALMI, appendicular lean mass index; BMR, basal metabolic rate; ECW, extracellular water; FFMI, Fat‐free mass index; GNRI, geriatric nutritional risk index; ht, height; LTMI, lean tissue mass index; pre‐ and post‐, pre‐ and post‐mid‐week haemodialysis session; SCI, simplified creatinine index; SLMI, soft lean mass index; SMMI, skeletal lean mass index; statin, 3‐hydroxy‐3‐methylglutaryl coenzyme‐A inhibitor; TBW, total body water.

In terms of sarcopenia, 68.7% met the HGS criteria for sarcopenia, 32.9% met the ALMI criteria and 27.7% met both criteria. As for frailty, 447 (43.7%) had CFS scores of ≥ 5 and were classified as frail. Applying the GNRI nutritional risk assessment, 5.6% were at major risk, 10.4% were at moderate, 13.9% were at low and 71.8% were not at risk.

Patients were divided into quartiles according to the HGS index, and there were proportionately more men, fewer diabetics and those of Asian ethnicity in the highest quartile (Table 1). In addition, patients in the highest quartile had less co‐morbidity, sarcopenia and frailty, and were more likely to undergo transplantation and have lower mortality. Dialysis urea clearance was slightly lower in this group. As expected, measured HGS was greater in the highest HGS index group, along with assessments of lean body mass, with lower ECW/TBW and higher ECW/height both pre‐ and post‐dialysis (Table 2). Although there were no differences in nutritional assessments, including SCI and GNRI, fewer patients were classified as being in the major risk GNRI group as the HGS index increased. There were no differences in standard laboratory investigations.

**Figure 1:**
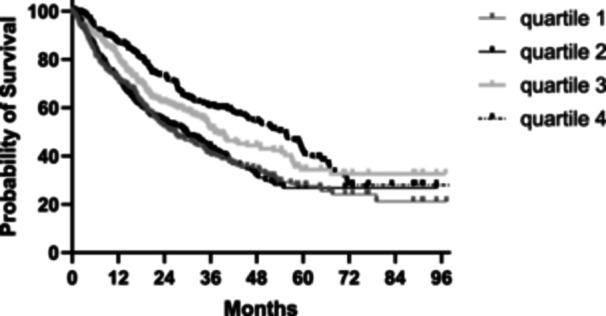
Kaplan–Meir log rank (Mantel–Cox) analysis demonstrated a significant difference in survival between the highest quartile (4) and lowest quartile (1) hand grip strength index quartiles. *χ* ^2^ = 24, *p* < 0.001.

On follow‐up, 22% had undergone transplantation and a total of 591 (57.8%) patients had died by May 2024, with 563 deaths (55%) after censoring for transplantation. The follow‐up of patients who remained dialysis‐dependent was for 22.3 (8.6–47.1) months. To investigate factors associated with mortality, using ROC analysis, mortality was associated with age, co‐morbidity and variables linked to muscle strength, muscle mass and physical ability, nutritional assessments and inflammation (Table 3). Kaplan–Meier log rank (Mantel–Cox) analysis showed a significant difference in survival between the higher and lower HGS index quartiles (Figure 1).

**Table 3:** Receiver operator curve (ROC) for survival censored for transplantation. Variables statistically associated with patient mortality.

Variable	ROC area	Standard error	95% Confidence limits	*p* value
Age (years)	0.799	0.014	0.772–0.826	< 0.001
ECW/TBW post‐HD	0.794	0.02	0.755–0.833	< 0.001
ECW/TBW pre‐HD	0.768	0.021	0.726–0.810	< 0.001
Clinical frailty score	0.759	0.022	0.716–0.801	< 0.001
SCI (mg/kg/day)	0.257	0.022	0.213–0.300	< 0.001
Serum creatinine (μmol/L)	0.298	0.024	0.309–0.405	< 0.001
Davies co‐morbidity	0.677	0.17	0.644–0.710	< 0.001
Hand grip strength (kg)	0.325	0.024	0.278–0.372	< 0.001
Lean tissue index (kg/m^2^)	0.326	0.024	0.281–0.371	< 0.001
Hand grip index	0.341	0.025	0.293–0.389	< 0.001
Albumin (g/L)	0.357	0.025	0.309–0.405	< 0.001
Geriatric nutritional risk index	0.38	0.018	0.351–0.414	< 0.001
Ethnicity	0.401	0.018	0.366–0.436	< 0.001
C‐reactive protein (g/L)	0.592	0.025	0.542–0.641	< 0.001
Dialysis vintage months	0.59	0.025	0.540–0.640	< 0.001
Sarcopenia (ALMI and HGS)	0.585	0.018	0.550–0.620	< 0.001
Serum urea (mmol/L)	0.422	0.026	0.372–0.472	0.002
Weight (kg)	0.447	0.026	0.397–0.498	0.004
Skeletal muscle mass index (kg/m^2^)	0.433	0.026	0.382–0.480	0.008

Abbreviations: ALMI, appendicular lean mass index; ECW, extracellular water; HGS, hand grip strength; pre‐ and post‐, pre‐ and post‐dialysis session; TBW, total body water.

Multivariable Cox survival models were then analysed using the variables in Table 3, and mortality was independently associated with age, dialysis vintage, ECW/TBW ratio, co‐morbidity, HGS index, C‐reactive protein (CRP), GNRI risk and ethnicity (Table 4), whereas SCI, sarcopenia, frailty and measures of body composition were all eliminated.

**Table 4:** Cox proportional survival model with patients censored at the time of transplantation.

Variable	*β*	StE *β*	Wald	OR	95% CI	*p* value
Age (years)	0.036	0.004	86.9	1.04	1.029–1.045	< 0.001
Davies co‐morbidity	0.215	0.042	26.2	1.24	1.142–1.347	< 0.001
ECW/TBW % post‐dialysis	0.142	0.029	24.5	1.15	1.089–1.219	< 0.001
Log dialysis vintage (months)	0.319	0.067	22.8	1.38	1.27–1.588	0.004
Hand grip strength index	−0.0076	0.002	11.4	0.99	0.991–0.998	< 0.001
Log C‐reactive protein (mg/L)	0.266	0.097	8.8	1.30	1.093–1.540	0.003
Geriatric nutritional index grade	0.112	0.052	4.7	1.12	1.010–1.259	0.031
Ethnicity	−0.118	0.057	4.5	0.89	0.795–0.993	0.038

*Note:* Multivariable Step backward Wald with variables statistically associated with mortality from Table 3, and eliminated if not statistically significant, unless improved model fit or demonstrating collinearity.

Abbreviations: 95% CI, 95% confidence interval; ECW, extracellular water; Ethnicity African, Afro‐Caribbean; OR, odds ratio; StE *β*, standard error of beta; TBW, total body water.

## 4 Discussion

Although there have been improvements in dialysis technology, patient mortality remains high and similar to that of some of the more common solid organ malignancies. Following the greater availability of dialysis services, increasing numbers of older patients with additional co‐morbidities and ESKD are now offered dialysis treatment in economically developed countries. As such, markers of muscle mass, strength, function and nutritional assessments have been reported to be key risk factors for predicting mortality [4, 10, 13, 22, 23]. Therefore, we investigated two of the newer proposed risk factors, the HGS index as an assessment of muscle function [16] and the SCI, a marker of nutritional intake [23], with standard traditional risk factors. Although both the HGS index and SCI were significantly associated with mortality on univariate analysis, only the HGS index was retained as an independent factor in multivariable models of mortality. Although the SCI equation includes adjustments for both gender and age, the SCI has been shown to be higher for both younger and male dialysis patients [13]. The SCI uses the single pool dialysis session urea clearance (spKt/V) and pre‐dialysis serum creatinine. We calculated the SCI based on a single dialysis session, and although we standardised measurements to the mid‐week dialysis session, there can be variations between dialysis sessions that could potentially alter these variables. Even so, on ROC analysis, the SCI was very strongly associated with mortality but was not retained in the multivariable model due to the stronger effects of patient age, co‐morbidity and other factors. A previous study from Japan reported an increased mortality for those with an SCI of < 20 mg/kg/day, and although there was a significant difference in mortality between those patients in the highest and lowest SCI quartiles in their analysis, there was also a major difference in age between the highest and lowest quartiles (53 vs. 75 years) [23]. For our own patients, taking a cut‐off of 20 mg/kg/day, the age of the higher SCI group was considerably younger (51.5 [40.7–58.7] vs. 71.9 [62.9–78.9] years, *p* < 0.001) and mortality was substantially lower (24.4 vs. 63.6%, *χ* ^2^ = 125, *p* < 0.001).

As the HGS index, a comparison between predicted and measured HGS, was retained in the multivariable survival models, we compared quartiles, and unlike the SCI, there were no major differences in patient age and laboratory investigations, although there were fewer diabetics, women and Asians in the highest quartile. Patients with greater co‐morbidity, sarcopenia, frailty and lower measurements of muscle mass and lower assessments of nutritional intake all had lower measured HGS compared to predicted HGS.

Apart from the HGS index, as expected, age, co‐morbidity, inflammation and longer prior dialysis treatment were all independent risk factors for mortality, in keeping with previous reports [24]. We also noted that nutritional assessment using the GNRI risk tool was also associated with increasing mortality, and the GNRI has been reported to be an effective tool for identifying haemodialysis patients with nutrition‐related risk for both all‐cause and cardiovascular disease [25]. In the United States, there are well‐described differences in survival between White and Black dialysis patients [26], whereas in the United Kingdom, despite greater social deprivation, Black ethnicity has been reported to be a survival advantage [27], although other studies showed no difference in survival between White, African and Asian haemodialysis patients after adjusting for age [28]. Our highest HGS index quartile had fewer Asians and more Whites, and this probably reflects the differences reported in terms of body composition, particularly skeletal muscle mass between patients from different ethnic backgrounds [29]. As expected, HGS was greater in male patients, and fewer men were in the lowest HGS index group compared to the highest quartile group. However, in keeping with many studies reporting on ESKD patients treated by dialysis, gender was not associated with mortality. However, we also found that the ratio of ECW/TBW was a risk factor for mortality, with higher values associated with mortality. Although ECW/TBW is associated with ECW volume overload, and volume overload is an established risk factor for mortality in dialysis patients [30], an increased ECW/TBW ratio can also be due to loss of intracellular water, and as muscle contains around 90% water, loss of muscle mass will also increase ECW/TBW [31]. In addition, inflammation by increasing endothelial permeability can also lead to an increased ECW/TBW ratio [32]. Comparing HGS index quartiles, prior to dialysis, there were no differences in ECW/TBW ratios, but post‐dialysis, the ECW/TBW ratio was higher in the lowest HGS index group, and as this group had a lower ECW/height ratio compared to the highest, suggesting that the difference in ECW/TBW ratios was not simply due to an increased ECW.

As with any observational study, one must consider potential confounders. Although we report on over 1000 dialysis patients, we only analysed HGS measurements at a single time point, along with body composition and laboratory measurements from a single dialysis session. Ideally, serial measurements and trends would be more useful in predicting patient trajectories and allowing evaluation of potential interventions [13]. Depending on the cause of the original kidney disease, previous organ transplantation and other co‐morbidities, kidney patients may be prescribed steroids. As such, 22% of patients had received one or more steroid doses and as patients were managed in other institutions, we do not have relevant information as to dosing and duration. At the time of the study, and follow‐up of the dialysis cohort, 6.8% were prescribed small doses of prednisolone. However, there was no difference in the historic or current prescription of steroids and measurements of HGS or the HGS index.

The demographics of the ESKD population treated by dialysis have changed over time in economically developed countries, with an increasing number of older co‐morbid patients now offered treatment. An increasing number of studies have reported that nutritional factors are associated with an increased risk for mortality, and some simple screening tools have been proposed to identify patients at greater risk. We investigated whether the SCI, a nutritional marker for dietary protein intake, and the HGS index, a comparison of measured HGS to expected HGS, were associated with mortality in ESKD dialysis patients. We found that the lower the HGS index, the greater the association with mortality. As more elderly co‐morbid ESKD patients are now being offered dialysis, simple rapid screening tests, such as the HGS index, are required to risk‐assess patients in routine clinical practice, to both earlier identify patients who may potentially require nutritional and or other support, but also to increase awareness and introduce realistic expectations for both patients and medical staff.

## Author Contributions

**Mai Nguyen:** Data collection and collation, and manuscript revision. **Andrew Davenport:** Formulation of the research idea, application of statistical analysis and revision of the final version to be published.

## Ethics Statement

This retrospective audit followed the UK National Health Service (NHS) guidelines for clinical audit and service development, and registered with the Royal Free Hospital audit department. development. As the audit complied with NHS guidelines (UK NHS guidelines for clinical audit and service development, available at http://www.hra.nhs.uk/documents/2013/09/defining-research.pdf, and http://www.gov.uk/government/publications/health-research-ethics-committees governance arrangements).

## Conflicts of Interest

The authors declare no conflicts of interest.

## Transparent Peer Review

The peer‐review history for this article is available at https://www.webofscience.com/api/gateway/wos/peer-review/10.1111/jhn.13414.

## Transparency Declaration

The lead author affirms that this manuscript is an honest, accurate, and transparent account of the study being reported; that no important aspects of the study have been omitted; and that any discrepancies from the study as planned (and, if relevant, registered) have been explained.

## Data Availability

All data generated or analysed during this study are included in this article. Further enquires can be directed to the corresponding author.
